# Association between stress and eating behaviour among Malaysian adolescents prior to examination

**DOI:** 10.1038/s41598-023-34699-3

**Published:** 2023-05-15

**Authors:** Nurfazlinda Md Shah, Nasrin Aghamohammadi, Nithiah Thangiah, Ai Kah Ng, Hazreen Abdul Majid

**Affiliations:** 1 Centre for Population Health (CePH), Department of Social and Preventive Medicine, Faculty of Medicine, University of Malaya, Kuala Lumpur, Malaysia; 2 Department of Nutrition, Faculty of Public Health, Universitas of Airlangga, Surabaya, Indonesia; 3 School of Chiropractor, AECC University College, Parkwood Campus, Parkwood Road, Bournemouth, BH5 2DF Dorset UK

**Keywords:** Diseases, Risk factors

## Abstract

Studies have shown the prevalence of mental health and obesity among adolescents is at increasing trend due to urbanisation and changes in lifestyle. This study is to investigate the level of stress and its impact on eating behaviour among Malaysian adolescents. A total of 797 multi-ethnic Malaysian secondary school student participated in this cross-sectional study. Data was collected two weeks prior to final year examination. A validated Cohen Perceived Stress Scale questionnaire was used to assess the stress level with subsample analysis of 261 participants’ saliva cortisol level. A validated Child Eating Behaviour questionnaire was used to explore eating behaviours. There were 29.1% adolescents having high stress with the mean saliva cortisol 3.8 nmol/L. A positive correlation was observed between perceived stress and emotional overeating; stronger among urban (r = 0.32), female (r = 0.31), underweight (r = 0.34) and moderately stressed adolescents (r = 0.24). In addition, a positive correlation was found between perceived stress and food responsiveness; strongest among Malay (r = 0.23), male (r = 0.24), underweight (r = 0.30) and adolescents with high perceived stress (r = 0.24). The perceived stress level prior to exam period affects the emotional eating and external eating patterns of adolescents.

## Introduction

Stress have become more prevalent due to urbanization and a hectic lifestyle. The proportion of global disability adjusted life-years (DALYs) attributable to mental disorders is 4.9% and the rate has not changed for the past 30 years^1^. Moreover, in Malaysia, prevalence of mental illness among children and adolescents aged 15 years and below has increased from 13% in 1996 to 20% in 2011^2^, with one in five Malaysian adolescents experiencing depression^3^. This is concurrent with an increasing prevalence of obesity among Malaysian children and adolescents aged 18 years and below from 6.1% in 2011 to 11.9% in 2015 and 14.8% in 2017^2–4^.

Persistent chronic stress negatively affect both physical and mental health^5^, leading to development of other mood disorders such as anxiety and depression which can directly or indirectly cause or contribute to the worsening of a huge number of diseases and chronic disorders such as hypertension, hypercholesterolemia and cardiac diseases^6–10^. This could be explained by the overall effect of stimulation of the Hypothalamic Pituitary Adrenal (HPA) Axis pathway following a stressful event. While the release of cortisol hormone stimulates the appetite and increases food intake, the release of other hormones through the sympathetic adrenal medullary pathway contributes to the development of non-communicable diseases^11^. Over the past few years, salivary cortisol has become the most popular biomarker in stress studies where it is utilized as an objective measurement of stress levels^12^. The saliva cortisol level is sometimes referred to as the measurement of ‘true stress’.

Studies suggested that stress can have a significant impact on eating behaviour^13–15^. A national survey conducted in America in 2007 reported that nearly half of the general population stated that they feel more stressed compared to 5 years ago, with 43% reporting that they utilized food as a coping mechanism to deal with stress^13^. A Malaysian study reported high levels of stress and unhealthy food-related behaviour patterns among young professional women ^14^. In addition, a study found that Malaysian adolescents who experienced high levels of stress were more likely to increase likelihood of unhealthy eating behaviors such as overeating, snacking, and consuming high-fat and high-sugar foods. The study also found that stress-related eating was a significant predictor of body mass index (BMI) among adolescents^15^. Also, a study conducted among Belgian children reported positive associations between emotional, peer, conduct and hyperactivity problems and the consumption of both sweet and fatty food, which implies that these problems contribute to the development of overweight in children^16^. Hence it has been postulated that stress-related eating is one of the factors contributing to the increasing prevalence of overweight and obesity.

The direct causes of obesity are still not well understood, but it is believed that obese individuals have a weaker response to internal signals of fullness and a stronger response to external food cues^17^. The emotional state of an individual is an important determinant of eating behaviour, but other factors, including internal and external determinants such as the availability of food, knowledge, attitudes and individual experience^18^, may also be at play. The increasing prevalence of obesity among Malaysian has triggered a sense of urgency among policy makers, stakeholders and researchers to identify the modifiable behaviours contributing to obesity. However, while there clearly a need to better understand the eating behaviour patterns of adolescents, the number of studies on the relationship between stress, eating behaviour and body composition among the adolescent population is very limited^13,14,19–23^. Yet, it is important that this group in particular remain healthy as the existence of an unhealthy future generation will have major socio-economic implications for the nation. Therefore, in light of the above, the first objective is to investigate the stress perceived by Malaysian adolescents and its impact on their eating behaviour. The second objective of this study was to examine the “true stress” using salivary cortisol. The results will provide valuable information that will assist in developing effective strategies to combat unhealthy eating behaviours and prevent the negative consequences of poor dietary habits among adolescents.

## Methods

### Study area and population

This study is a cohort study conducted as part of an ongoing larger cohort study, the Malaysian Health and Adolescents Longitudinal Research Team (MyHeART) Study^24^. The MyHeART study has been recruiting public secondary school students in Malaysia from three states in Peninsular Malaysia (namely Selangor, Kuala Lumpur and Perak) since 2012. Northern Region represented by state of Perak and Central Region consists of Kuala Lumpur and Selangor. All government-funded secondary schools were included in the study except vernacular schools, vocational schools, boarding schools, religious schools, private schools and schools for children with disabilities. This is because the government-funded secondary schools are the main secondary school stream in the country. Besides, the syllabus, curricular activities and the rules governing the government-funded secondary schools may affect the stress level of the students differently than those excluded schools. This study involved participants aged 16 years old. D Cortisol study were conducted during the 2 weeks prior to the final-year school examination.

### Sample size calculation

The sample size to determine the stress level and eating behaviour patterns among adolescents was calculated by using G*Power 3.1.9.2 software. The effect size was calculated based on the findings by Groesz et al.^13^ which used partial correlation to determine the relationship between stress and all eating behaviour indices after controlling for age, BMI, education and income. The correlation of bivariate normal model and type of power analysis were selected under the statistical exact test family. Alpha level of 0.05 was taken at the power of 80%. The effect size of the association between perceived stress and palatable non-nutritious food intake shown the largest sample size (518 subjects). Therefore, the sample size required for the study was 518 subjects. However, a sample size of 783 subjects was selected in order to accommodate a non-response rate of 51% as encountered in the initial recruitment of the MyHeART cohort.

In this study, second objective is to measure stress level using objective measure in which is saliva cortisol. Due to budget constraints, the saliva cortisol analysis was not conducted for whole sample (n = 783 subjects). Therefore, researchers only conducted the analysis on subsample of 783 subjects. The subsample size for the saliva cortisol analysis was calculated based on the prevalence rate of psychiatric morbidity among children and adolescents below 15 years old as reported in NHMS IV^2^. The minimum sample size required for the subsample element of the study was 188. However, a sample size of 245 subjects was chosen in order to accommodate a non-response rate of 30%. All calculations are with an alpha of 0.05 and power of 80%.

### Sampling

Random cluster sampling was performed on 15 schools involved in the MyHeART study. As a result, 11 schools were selected as clusters; seven schools from urban areas and four schools from rural areas. Then, systematic random sampling techniques were used to determine the sample size of the subsample population from among these schools for the saliva sample collection. Prior to data collection, approval was obtained from respective departments and schools. Parental informed consent form and research information sheet were distributed to all the prospective participants and informed consents were obtained from both parents and participants prior to data collection process.

### Data collection

Three data collection methods were utilized in this study: a set of questionnaires, anthropometric measurement and saliva sampling.

(i) Questionnaires

Data were collected from the participants by using a set of three self-administered questionnaires: the Socio-Demographic Background Questionnaire, Cohen Perceived Stress Scale (CPSS) questionnaire and Child Eating Behaviour Questionnaire (CEBQ). The participants completed all three self-administered questionnaires without guidance from the researcher within approximately 30 min.

- The socio-demographic background questionnaire

This questionnaire was developed by the researcher to obtain demographic data and possible confounding factors, including individual factors, family factors and environmental factors. The questionnaire was reviewed by an expert panel, finalized and then pre-tested in both the English and Malay language on 56 adolescents visiting the Adolescent Centre at the National Population and Family Development Board Headquarters in Kuala Lumpur prior to its usage in the actual study.

(b) CPSS questionnaire

The CPSS questionnaire contains 10 items and is a self-report measure of stress, which has been validated for use among Malaysian adolescents^25^*.* The participants respond to each question by giving a score on a five-point Likert scale ranging from 0 (never) to 4 (very often) to indicate how often they have experienced a stressful event within the past 1 month. The total score ranges from 0 to 40. Higher scores indicate greater perceived stress. Six items are negatively stated to represent ‘perceived distress’, whereas four items are positively stated to represent ‘perceived coping’. Permission was obtained from the author of the questionnaire prior to its usage in this study.

The CPSS score was used to classify the participants into three stress level categories: low, moderate and high perceived stress. The low perceived stress category included participants with a CPSS score of 13 and below. Participants with a score of 14–26 were classified as belonging to the moderate perceived stress level category and participants with a score of 27 and above were classified into the high perceived stress level category^26^.

(c) CEBQ questionnaire

The original CEBQ consisted of 35 items^18^ and was used to measure eating behaviour among children below 12 years old. The self-reported CEBQ was later validated among Malaysian adolescents^27^. Permission to use the Malaysian validated version of the questionnaire was obtained from the authors prior to usage. The CEBQ used in this study consisted of eight eating behaviour items representing three eating patterns: (I) Emotional Eating [two items: (i) emotional overeating (EOE); (ii) emotional undereating (EUE)]; (II) Externally Induced Eating [three items: (i) food responsiveness (FR); (ii) enjoyment of food (EOF); (iii) desire to drink (DD)]; and (III) Restrained Eating [three items: (i) satiety responsiveness (SR); (ii) slowness in eating (SE); (iii) food fussiness (FF)]. Emotional eating indicates that the subject eats in response to emotions such as nervousness, happiness or excitement, whereas externally induced eating indicates that eating is in response to stimuli such as the smell and taste of food. Restrained eating indicates that the subject exerts control over their eating and tries to refrain from eating. The response options in this questionnaire are based on a five-point Likert scale and range from 1 (never) to 5 (always)^18^.

(ii) Anthropometric measurement

The body composition measurements of each participant were taken by a trained researcher using calibrated equipment. The height of the participants was taken without socks, shoes and without hair ornaments, and recorded to the nearest 0.1 cm by using a vertical portable stadiometer (Seca 217, UK). Their weight were measured with students barefoot and wearing either school uniform or t-shirts and tracksuits, and recorded to the nearest 0.1 kg by using a digital electronic scale, the portable body composition analyser (Tanita SC-240, Netherlands).

(iii) Salivary biomarkers

Saliva samples were collected from the subsample of participants once per visit; approximately at 10 am. Collection of the saliva was performed by the students themselves after a brief explanation by the researcher. Each participant clean their mouth before passively drooling approximately 1 ml of saliva into a given sample tube, assisted by saliva collection aid provided. The time and date of sample collection were recorded and the samples were labelled. The saliva samples were then stored in a frozen form below minus 80 °C in the Department of Social and Preventive Medicine at the University of Malaya prior to analysis. The samples were analysed by using a Saliva Cortisol Enzyme Immunoassay Analysis Kit purchased from Salimetrics Europe Ltd (UK). A total of seven analyses were conducted rigorously by following the protocol guideline (Protocol No: 1-3002 (5PK 1-3002-5)) and the results were read using a microplate reader, Infinite 200 Pro Multiple Reader supplied by Tecan Group Ltd (Mannedorf, Switzerland).

### Statistical analysis

Data were analysed using IBM SPSS Statistics 24.0 (United States). Descriptive statistics obtained by using univariate approaches were used to describe the study population and measure the variance in the participants’ socio-demographic data. Descriptive statistics were also used to determine the prevalence and distribution of stress and eating behaviour patterns of the adolescents. The Shapiro–Wilk test of normality was used to test the normality of the data distribution. The results were expressed as mean and 95% confidence interval (CI) and as number of subjects and a percentage. An independent-sample T-Test and Chi-square test were used to analyse the variations in the continuous and categorical variables, respectively. Analysis of variance was used to determine differences in eating behaviour and stress level across the demographic categories, obesity categories and stress level categories. Pearson’s correlation analyses were used to determine the strength of the relationships between groups of selected variables. The results analysis used an alpha level of 0.05 and a 95% CI and a p-value. A p-value of less than 0.05 was considered statistically significant.

### Ethics approval

This study was conducted according to the guidelines laid down in the Declaration of Helsinki and all procedures involving human subjects were approved by the Medical Ethics Committee of the University of Malaya Medical Centre (Ref No: 896.34). Written approval was also obtained from the Ministry of Education, Malaysia, the respective Education Department of the states of Perak, Kuala Lumpur and Selangor, and the principals of the schools involved in the study.

## Results

Of the 802 respondents, 797 were included in the final analysis. Out of the subsample of 265 participants in the saliva collection, 261 samples were included in the final analysis. The socio-demographic characteristics of the participants are shown in Table 1.

**Table 1:** Prevalence and distribution of stress among the adolescents based on Cohen Perceived Stress Scale Score according to stress level categories.

	Total [n (%)]	Stress level category			Pearson Chi-Square
		Low	Moderate	High	
Overall	797 (100)	72 (9.0)	493 (61.9)	232 (29.1)	
Gender					
Male	317 (39.8)	38 (12)	212 (66.9)	67 (21.1)	χ^2^ = 18.72
Female	480 (60.2)	34 (7.1)	281 (58.5)	165 (34.4)	p = < 0.01*
School location					
Urban	482 (60.5)	38 (7.9)	287 (59.5)	157 (32.6)	χ^2^ = 7.87
Rural	315 (39.5)	34 (10.8)	206 (65.4)	75 (23.8)	p = 0.02*
Ethnicity					
Malay	597 (74.9)	53 (8.9)	366 (61.3)	178 (29.8)	χ^2^ = 7.83
Chinese	93 (11.7)	7 (7.5)	54 (58.1)	32 (34.4)	p = 0.25
Indian	69 (8.7)	9 (13.0)	49 (71.0)	11 (15.9)	
Others	38 (4.8)	3 (7.9)	24 (63.2)	11 (28.9)	
BMI category					
Obese and overweight	210 (26.3)	19 (9.0)	120 (57.1)	71 (33.8)	χ^2^ = 3.30
Normal weight	355 (44.5)	33 (9.3)	224 (63.1)	98 (27.6)	p = 0.51
Under weight	232 (29.1)	20 (8.6)	149 (64.2)	63 (27.2)	

BMI, body mass index Scores ranging from 0 to 13 would be considered low stress: 0–13; moderate stress: 14–26 and high stress: 27–40.

*p < 0.05, **p < 0.01.

The individual, environmental and family factors investigated in this study are presented in Table 2 by school location.

**Table 2:** Individual, family and environmental factors of participants according to school location.

		Urban n (%)	Rural n (%)	Total n (%)
Individual factors				
BMI category	Overweight and obese	133 (27.6)	77 (24.4)	210 (26.3)
	Normal weight	226 (46.9)	129 (41.0)	355 (44.5)
	Underweight	123 (25.5)	109 (34.6)	232 (29.1)
Academic achievement	Good	31 (6.5)	10 (3.2)	41 (5.2)
	Average	114 (23.9)	62 19.9)	176 (22.3)
	Below Average	332 (69.6)	240 (76.9)	572 (72.5)
Financial autonomy	High	290 (60.5)	68 (21.8)	358 (45.2)
	Medium	175 (36.5)	238 (76.0)	413 (52.1)
	Low	14 (2.9)	7 (2.2)	21 (2.7)
Attempts to reduce weight	Yes	283 (59.2)	173 (56)	456 (57.9)
	No	195 (40.8)	136 (44)	331 (42.1)
Eat to cope with stress	Yes	161 (33.6)	117 (37.9)	278 (35.3)
	No	318 (66.4)	192 (62.1)	510 (64.7)
Family factors				
Father’s education	Not known	108 (22.4)	166 (20.8)	58 (18.4)
	Tertiary	120 (24.9)	153 (19.2)	33 (10.5)
	Secondary	210 (43.6)	401 (50.3)	191 (60.6)
	Primary and below	44 (9.2)	77 (9.7)	33 (10.5)
Mother’s education	Not known	99 (20.5)	156 (19.6)	57 (18.1)
	Tertiary	109 (22.6)	132 (16.6)	23 (7.3)
	Secondary	229 (47.5)	425 (53.3)	196 (62.2)
	Primary and below	45 (9.3)	84 (10.5)	39 (12.4)
Parents’ marital status	Married	413(85.7)	687(86.2)	274(87.0)
	Single parent	69(14.3)	110(13.8)	41(13.0)
Regard parents as role model	Yes	384(80.7)	652(83.1)	268(86.7)
	No	92(19.3)	133(16.9)	41(13.3)
Meal with family	Frequently	206 (43.2)	321 (40.5)	115 (36.5)
	Sometimes	265 (55.6)	461 (58.2)	196 (62.2)
	Never	6 (1.3)	10 (1.3)	4 (1.3)
Environmental factors				
Opinion on canteen food	Nutritious	36 (7.5)	14 (4.4)	50 (6.3)
	Not Nutritious	246 (51.0)	138 (43.8)	384 (48.2)
	Not Sure	200 (41.5)	163 (51.7)	363 (45.5)
Presence of junk food vendor outside school compound	Yes	338 (70.1)	141 (45.0)	479 (60.3)
	No	84 (17.4)	85 (27.2)	169 (21.3)
	Not Sure	60 (12.4)	87 (27.8)	147 (18.5)
Bought junk food from vendor	Frequently	13 (2.7)	4 (1.3)	17 (2.1)
	Sometimes	288 (59.8)	184 (58.8)	472 (59.4)
	Never	181 (37.6)	125 (39.9)	306 (38.5)
Fast food consumption	Frequently	35 (7.3)	8 (2.5)	43 (5.4)
	Sometimes	439 (91.1)	298 (94.6)	737 (92.5)
	Never	8 (1.7)	9 (2.9)	17 (2.1)

### Stress among Malaysian adolescents

The CPSS scores and saliva cortisol levels are shown in Table 3. The mean stress score was 18.7 (95% CI 18.4, 19.0) and was significantly higher among students who were female, urban and below average in terms of academic achievement. The mean saliva cortisol level was 3.8 nmol/L (95% CI 3.4, 4.3). No significant difference in the stress score and saliva cortisol level was observed in relation to body mass index (BMI) category and pocket money.

**Table 3:** Stress among the adolescents based on Cohen Perceived Stress Scale score and saliva cortisol level.

Variables	n	Cohen perceived stress scale [mean score (95% CI)]		Saliva cortisol level (nmol/L) [mean level (95% CI)]		
Total	797	18.7 (18.4, 19.0)	p	261	3.8 (3.4, 4.3)	p
Gender						
Male	317	17.9 (17.4, 18.3)	< 0.01*	100	4.3 (3.5, 5.1)	0.08
Female	480	19.3 (18.9, 19.6)		161	3.5 (3.0, 4.0)	
School type						
Urban	482	19.1 (18.7, 19.5)	< 0.01*	152	3.9 (3.3, 4.4)	
Rural	315	18.1 (17.7, 18.6)		109	3.8 (3.0, 4.5)	0.84
Ethnicity						
Malay	597	18.8 (18.5, 19.2)	0.02*	193	3.5 (3.0, 3.9)	0.03*
Chinese	93	19.2 (18.5, 19.9)	*Mal-Ind	34	4.5 (3.0, 5.9)	*Ind-Mal
Indian	69	17.3 (16.3, 18.3)	*Chi-Ind	22	5.7 (3.4, 8.0)	
Others	38	18.3 (17.1, 19.5)		12	4.3 (1.3, 7.3)	
BMI category						
Obese and overweight	210	19.0 (18.4, 19.5)	0.50	79	3.5 (2.8, 4.2)	0.18
Normal weight	355	18.7 (18.3, 19.1)		111	3.6 (3.0, 4.2)	
Underweight	232	18.5 (18.0, 19.1)		71	4.5 (3.4, 5.6)	
Academic achievement						
Good	41	18.4 (16.9, 20.0)	< 0.01*	10	5.9 (2.5, 9.4)	0.13
Average	176	17.9 (17.3, 18.5)	*Av-Below Av	61	3.5 (2.7, 4.3)	
Below average	572	19.0 (18.7, 19.3)		187	3.8 (3.3, 4.3)	
Pocket money						
High	358	18.9 (18.5, 19.4)	0.12	115	3.9 (3.3, 4.4)	0.96
Moderate	413	18.5 (18.1, 18.9)		139	3.8 (3.2, 4.5)	
Low	21	19.9 (17.8, 22.0)		4	3.3 (0.8, 5.8)	
Eating as stress coping strategy						
Yes	278	19.7 (19.1, 20.2)	< 0.01*	84	3.2 (2.6, 3.9)	0.08
No	510	18.2 (17.8, 18.5)		172	4.1 (3.5, 4.7)	

*Mal* Malay, *Ind* Indian, *Chi* Chinese, *Av* Average, *BMI* body mass index.

*p < 0.05, **p < 0.01.

The prevalence and distribution of stress among the adolescents according to the above mentioned perceived stress level categories is shown in Table 1. The prevalence of high perceived stress among the adolescents was 29.1%, and was significantly higher among female (34.4%) and urban (32.6%) adolescents. There was no significant correlation between perceived stress and saliva cortisol level (p = 0.209).

### Stress and eating behaviours

The results of the Pearson’s correlations between stress and eating behaviour among the adolescents are shown in Table 4. The strongest positive significant correlation was observed between perceived stress and EOE (r = 0.30) followed by FR (r = 0.22). No significant correlation was observed between the saliva cortisol level and all the eating behaviour items. Therefore, only the correlation between perceived stress and eating behaviour items was subjected to further analysis by gender, school location, ethnicity, BMI category and stress level category. Significant positive correlations between perceived stress and EOE were strongest among participants who were female (r = 0.31), urban (r = 0.32), underweight (r = 0.34) and had a moderate perceived stress level (r = 0.24). Positive significant correlations observed between perceived stress and FR were strongest among students who were male (r = 0.24), rural (r = 0.23), underweight (r = 0.30) and had high perceived stress (r = 0.24).

**Table 4:** Correlation between stress and eating behavior of the adolescents by gender, location, ethnicity, BMI category and stress level category.

Stress	n	Eating behavior score at baseline							
		Emotional eating		Restrained eating			Externally induced eating		
		EOE	EUE	SR	SE	FF	DD	EOF	FR
Cortisol level	228	− 0.06	− 0.01	− 0.03	0.01	− 0.02	0.03	− 0.06	− 0.04
CPSS score	592	0.30**	0.16**	0.19**	0.12**	0.07	0.09*	0.12**	0.22**
Gender									
Male	200	0.22**	0.27**	0.21**	0.17*	0.07	0.08	0.08	0.24**
Female	392	0.31**	0.08	0.15**	0.10*	0.05	0.10*	0.10*	0.20**
Location									
Urban	334	0.32**	0.12*	0.20**	0.09	0.10	0.11*	0.17**	0.21**
Rural	258	0.28**	0.20**	0.17**	0.15*	0.01	0.05	0.05	0.23**
Ethnicity									
Malay	439	0.29**	0.17**	0.18**	0.11*	0.07	0.13**	0.13**	0.23**
Chinese	69	0.31**	0.14	0.13	0.22	− 0.08	− 0.09	− 0.01	0.08
Indian	56	0.28*	0.09	0.33*	0.09	0.15	− 0.05	0.09	0.10
Others	28	0.10	− 0.02	0.15	0.38*	0.16	− 0.15	− 0.07	0.26
BMI category									
Overweight and obese	151	0.31**	0.16	0.28**	− 0.00	0.09	− 0.01	0.09	0.17*
Normal weight	267	0.28**	0.06	0.13*	0.21**	0.08	0.08	0.13*	0.20**
Underweight	174	0.34**	0.28**	0.19*	0.10	0.04	0.17*	0.16*	0.30**
Stress category									
High	167	0.18*	0.05	0.13	0.06	− 0.01	0.03	0.10	0.24**
Moderate	374	0.24**	0.11*	0.13*	0.14**	0.16**	0.10	0.12*	0.11*
Low	51	0.09	0.20	0.24	0.16	0.00	0.07	− 0.03	0.15

*EOE* emotional overeating, *EUE* emotional undereating, *SR* satiety responsiveness, *SE* slowness in eating, *FF* food fussiness, *DD* desire to drink, *EOF* enjoyment of food, *FR* food responsiveness, *CPSS* Cohen perceived stress scale, *BMI* body mass index.

*p < 0.05, **p < 0.01.

## Discussion

The results of this study reveal the magnitude of the stress problem among Malaysian adolescents prior to their final-year school examination and the significance of the impact of stress on their eating behaviours.

### Perceived stress among Malaysian adolescents

In general, this study found that Malaysian adolescents have a moderate perceived stress level, as reflected by the CPSS score of 18.7 during the near examination period. This study also found that prior to the national school examination, the prevalence of a high perceived stress level among Malaysian adolescent was 29.1% while 61.9% perceived that they were experiencing a moderate level of stress.

A local study reports that 38.5% of Malaysian school students have stress symptoms^28^. In 2019, another local survey reported that 424,000 children had mental health problem, in which 9.5% were adolescents between 10 to 15 years old^29^. Another study found a high prevalence of distress among secondary school students (32.8%) and the major stressor was identified as being academic-related issues^3,30^. An earlier study on stress, coping and social support that focuses on Malaysian students found that adolescents generally consider their life to be stressful^31^. Not only that, students aged between 12 to 13 years old had the highest rates of suicidal intention (11.2%) as reported in National Health and Morbidity Survey (NHMS) 2017^3^. The abovementioned studies have identified the magnitude of stress that exists in the student population and that it is as an important issue that needs to be addressed. However, this study is the first to identify the level of stress perceived by the adolescents themselves. This information is very alarming as it means that one out of three adolescents perceive that they are highly stressed and, conversely, only one out of 10 adolescents perceive that they have a low level of stress.

The prevalence of high perceived stress revealed in this study is significantly higher among female students. This is in line with some earlier findings in Malaysia which reported that stress are significantly higher among female adolescents^3,8,32,33^. Worldwide, sex differences in the incidence and severity of stress suggest that female tend to be more vulnerable in terms of their responses to stress^34^. This study also found that urban students perceive their level of stress to be higher as compared to rural students. It is likely that both individual and family factors have influenced this result. A greater proportion of the urban participants in this study have a higher level of academic achievement compared to the rural participants. Also, a higher number of urban parents have a higher education level than their rural counterparts. It is possible that being more educated and thus more aware of the importance of education could cause urban parents to have higher expectations regarding their children’s achievement and thus place extra pressure on their children as compared to rural parents. This is in line with the findings of a survey in 2013 that identifies that high expectations of parents in regards to their children achieving a level of academic excellence is one of the significant stress risk factors for Malaysian adolescents^8^. Another Malaysian study reports that 77% students perceive academic expectations as an important stressor^31^. The two studies however did not compare between the urban and rural populations as this study did. As for the influence of BMI on perceived stress, this study did not find any significant association despite a higher CPSS score being reported among the obese and overweight adolescents.

The findings of this study thus identify the segment of the target population that needs the most consideration when developing intervention programmes to promote mental health among adolescents.

### ‘True stress’ among Malaysian adolescents

The mean saliva cortisol level among the adolescents in this study was within the morning range at 3.8 nmol/L (95% CI 3.4, 4.3). Pennsylvania State University Behavioral Endocrinology Laboratory reports that following the circadian rhythm of cortisol, the normal morning reading of the saliva cortisol level among European adolescents aged 12 to 18 years old ranges between 0.58 to 24.4 nmol/L whereas the evening reading is lower and ranges from not detected to 7.1 nmol/L^35^. The level rises independently of the circadian rhythm in response to stress^36^.

A previous study conducted in a neighbour country, Singapore reports that students who rate that they are experiencing higher stress before examinations have increased levels of salivary cortisol^37^. Numerous previous studies have been conducted in Malaysia specifically to identify the magnitude of stress in the population and have revealed that it as an important issue that needs to be addressed^28–32,38,39^. However, none of these studies have used a biomarker as an objective measurement to support their results. To the best of our knowledge, there have been no other studies on the saliva cortisol level among Malaysian adolescents in school setting prior to the publication of the current study and therefore no within-country comparisons could be made. A western study conducted among American college students by Sladek et al. with a mean age of 18.9 years old reports a mean cortisol level of 6.69 nmol/L^40^. However, college and school population are not comparable as they differ in terms of study syllabus, curricular activities and study environment. Furthermore, the study had an earlier saliva collection time of 1 h after waking up from sleep. Also, the study by Sladek et al. has a limitation in that it has a much smaller sample size (n = 70) as compared to this study (n = 797).

A significant difference in the saliva cortisol level was only observed in terms of ethnicity, where Indian students were found to have a significantly higher mean saliva cortisol than Malay students. This finding does not correlate well with the results of perceived stress, where Indian students have significantly lower perceived stress than Malay students. No other similar studies in Malaysia were available to enable deeper exploration of between ethnicity comparisons.

The non-significant correlation between salivary cortisol level and perceived stress level is probably due to the variation between the students’ perception of stress and the ‘true stress’ they actually experience, which is reflected by the saliva cortisol level. Identifying stress among adolescents who are in a complex transitional stage and not always able to fully express themselves may be difficult and may cause even the health professionals to miss early signs of psychological illnesses that warrant intervention. Therefore, using the saliva cortisol level, a stress biomarker, as an indicator could be very helpful in identifying at-risk adolescents. However, despite the ability of cortisol as an important biomarker to help identify stress among adolescents, it should not be used as the sole measurement of stress as it could be influenced by numerous other factors such as smoking, caffeine intake, alcohol consumption and glycaemic load. Thus, integrating the usage of cortisol carefully with a subjective measurement of stress would be the most ideal approach.

### Association between stress and eating behaviour among Malaysian adolescents

A positive significant correlation was observed between perceived stress level and all the eating behaviour items except for food fussiness. Besides, a stronger positive significant correlation was observed between perceived stress level and EOE among female (r = 0.31), urban (r = 0.32) and underweight (r = 0.34) students.

The correlation was strongest between perceived stress and emotional overeating (r = 0.30) followed by perceived stress and food responsiveness (r = 0.22). This study also discovered a significant positive correlation between perceived stress and EOE among adolescents with both high and moderate perceived stress but not among those with low perceived stress. These findings are similar to those of some western studies conducted among adolescents which report that perceived stress is associated with EOE^41,42^. Furthermore, a cross-sectional study among Malaysian adolescents aged 18–25 years old also reports a strong significant correlation between stress and EOE (r = 0.43) and between stress and external eating pattern (r = 0.28)^43^. However, a study of stress and eating behaviour among Malaysian university graduates reports contradictory findings that stress does not affect uncontrolled eating and EOE^44,45^.

In this study, as no significant correlation between saliva cortisol level and any of the eating behaviour items was found, only the correlation between perceived stress level and eating behaviours were examined in relation to the effects of gender, school location, ethnicity and BMI category. A stronger positive significant correlation was observed between perceived stress level and EOE among female (r = 0.31), urban (r = 0.32) and underweight (r = 0.34) students. The finding is coherent with some studies conducted in the western countries which had reported that stress-related eating and emotional eating behaviour is more common in girls as compared to boys^19,41^.

This study also found that urban adolescents have a higher awareness and knowledge of healthy eating. However, they eat less healthily compared to rural students especially when dealing with stress, as reflected by the stronger correlation between stress and emotional overeating among these students. The stresses of urban life could be one of the factors that influence urban adolescents to eat unhealthily, which results in a higher prevalence of overweight and obesity among them than among rural adolescents. A higher prevalence of overweight among urban than rural adolescents has also been reported in a previous study on 200 respondents from two districts in the state of Selangor in Malaysia^46^. However, an earlier finding derived from the MyHeARTs’ cohort which have a larger sample size (n = 1361) involving three states in Malaysia had reported that Malaysian adolescents from rural areas are at higher risk of developing non-communicable diseases that is related to obesity such as type 2 diabetes and cardiovascular diseases compared to their urban counterparts^24^. This study is a sub cohort of the MyHeARTs’ cohort. Thus, the difference in the findings of the similar population could actually reflect the changes in the lifestyle of the adolescents as they grow older; comparing the adolescents when they were 13 years old during MyHeARTs study in 2012 and 16 year old of age in this study. Besides, it also found that the trend of dietary intake of the MyHeARTs’ cohort start to established once the adolescents reached the age of 15 years old^47^. Children’s eating behaviours at a younger age could be influenced more by the family environment, whereas at an older age, food choice decisions could be made by the adolescents themselves.

This study also found that the majority of urban parents have a better level of education, but it should not be assumed that such parents ensure that their children eat a healthy diet. For instance, a study found a high intake of fast food among children of mothers with a higher educational level^48^. This is supported by the findings of this study, where urban students, despite being high achievers and having parents with a higher education level, report more frequent fast-food consumption and the purchasing of food from junk food vendors. The changing lifestyle in urban areas has encouraged the rise of the eating out phenomenon in Malaysia, which is further aggravated by the higher financial autonomy and greater food availability in the city. Higher education allows individuals to have higher paid occupations, which results in higher financial autonomy. Higher perceived stress, higher financial autonomy and greater availability to unhealthy food could be factors influencing urban students to eat unhealthily as compared to rural students^49^.

In turn, this study found that more rural students eat to cope with stress as compared to their urban counterparts. Interestingly, students who admitted to eating to cope with stress have a significantly higher perceived stress level compared to those who do not use this coping strategy indicating that this coping strategy does not help to reduce stress. The practice of eating to cope among rural students could be due to a lack of awareness about what constitutes a healthy lifestyle as the majority of the rural students and their parents in this study have a lower level of academic achievement than urban students and their parents. It has been reported that mothers with a low level of education could affect their children’s intake by providing diet with not much thought of the nutrition contents and thus contributed to a higher BMI status^50^. However, this could not be the only reason of poor eating habit among the rural adolescents as their eating habit could be strongly influenced by rural students’ lower financial autonomy and by less food availability, and thus a higher percentage of rural adolescents are still in the underweight category compared to urban students. Both external forces in the urban and rural environment and internal drivers at home could thus contribute to the current trend of behaviour in food taking among the adolescents.

As regards the influence of the BMI category, this study found that an increase in the perceived stress level tends to increase the EOE and FR score of underweight participants more than it does those in the obese and overweight category. However, restrained eating behaviour, as shown by the correlation between perceived stress and the SR score, seems to be better among the obese and overweight participants. This finding does not support that of an earlier study which reports that adolescents under moderate or severe stress have a significantly higher BMI and consume more sweet and high-fat foods compared to adolescents with mild stress^51^. Also, another study had reported that, compared with non-stress-driven eaters, stress-driven eaters have a higher prevalence of overweight, obesity and abdominal obesity^19^. Besides, a study among university students also indicated that those who are not having high stress tends to consume lower energy, carbohydrate and sugar compared to those who are having stress under academic stress condition^52^. Thus, the findings revealed by this study need to be explored further because the use of self-administered questionnaires could have led to the possibility of underreporting among the obese and overweight students due to a fear of stigmatization and probably denial. The majority of obese and overweight adolescents also did not admit to utilize eating as a coping strategy when coping with stress. Such contradictory findings in different regions could be caused by cultural differences in a multi-ethnic Asian settings as the influence of culture is complex and mediated by biological, demographic, psychosocial and environmental factors^53^. It is interesting also to note boys who had shown depressive symptoms had long term concentrations of cortisol in hair^54^.

### Strengths and limitations of this study

The strength of this study lies in the method used to measure the stress level which employed both subjective and objective measurements. The study also analysed a fairly large sample size and covered three major states in Malaysia, which gives power to the study. The main limitation of this study was the inability to apply the objective measurement of stress to all participants and repeated daily measurements due to cost constraints. However, while the saliva cortisol analysis was conducted only on a subsample, it still had sufficient power. Another limitation is that potential underreporting may have occurred because the study collected data via self-reported questionnaires. Also, this study was only able to capture data on school-going adolescents. A longitudinal study on this subject would be able to better assess the effect of stress on eating behaviour and body composition among adolescents.

## Conclusions

The majority of Malaysian adolescents perceive that they have a moderate level of stress prior to school examinations when using Cohen Perceived Stress Scale Questionnaire (subjective measure). There is no association between perceived stress (subjective measure) and saliva cortisol level (objective measure). It appears that cortisol is associated in detecting acute stress and the perceived stress questionnaire is more useful in determining chronic stress. The perceived stress level affects the emotional eating and external eating patterns of adolescents. This study highlights the importance of combating unhealthy eating behaviours by incorporating a mental health module in the national health policy as early specifically for children and younger adolescents. A holistic approach involving the family, school and relevant authorities will help ensure the well-being of future generations.

## Data Availability

All information are incorporated into the article. Participant data is not publicly available.
